# Estimated intestinal absorption of phosphorus and its deposition in chosen tissues, bones and feathers of chickens receiving chromium picolinate or chromium nanoparticles in diet

**DOI:** 10.1371/journal.pone.0242820

**Published:** 2020-11-25

**Authors:** Anna Stępniowska, Krzysztof Tutaj, Aleksandra Drażbo, Krzysztof Kozłowski, Katarzyna Ognik, Jan Jankowski

**Affiliations:** 1 Department of Biochemistry and Toxicology, Faculty of Animal Sciences and Bioeconomy, University of Life Sciences in Lublin, Lublin, Poland; 2 Department of Poultry Science, Faculty of Animal Bioengineering, University of Warmia and Mazury, Olsztyn, Poland; Tokat Gaziosmanpasa University, TURKEY

## Abstract

The aim of the study was to determine whether the level and form of Cr in the diet of chickens influences its accumulation in tissues as well as intestinal absorption of P and its deposition in tissues. The experiment was carried out on 405 one-day-old male Ross 308 chickens that were randomly divided into five treatment groups. Control group was fed the diet without supplemental chromium; experimental groups were fed the diet with 3 or 6 mg/kg chromium picolinate (Cr-Pic) and with 3 or 6 mg/kg chromium nanoparticles (Cr-NP). Chromium was found to accumulate in the tissues of the ileum, liver, breast muscle, bones skin and in feathers of chickens. Chromium deposited in the ileum of chickens does not affect the ex vivo estimated intestinal absorption of P. The use of Cr in the diet of chickens carries the risk of lowering P levels in femur.

## Introduction

Dietary chromium (Cr) affects birds physiology, improves carbohydrate and lipid metabolism, reduces stress responses, stimulates the immune systems, and above all can improve production results and reduce carcass fat [1–7]. Although Cr is not currently considered as essential trace element for poultry, researchers are increasingly investigating its potential to relieve heat stress. Moreover, commercial Cr-based preparations exhibiting this effect are already available among feed additives. Toxicity of Cr depends on its valance state, Cr(VI) is more toxic than Cr(III). However, according to Bagchi et al. [8] Cr(III) in high doses cause renal impairment, anemia, hemolysis, tissue edema, liver dysfunction; neuronal cell injury, enhanced production of hydroxyl radicals, chromosomal aberration, depletion of antioxidant enzymes, and DNA damage. Maximum Residue Limits (MRL) are established for residues of feed additives or veterinary medicines. Due to the lack of sufficient data for Cr(III), the MRL for tissues has not been determined for its administration by either the oral or inhalation route [9]. There are no National Research Council (NRC) [10] recommendations for Cr in poultry, but this organization recommends 300 μg/kg of Cr in the diets of laboratory animals. The European Food Safety Authority (EFSA) [11] has not yet established maximum dosages for various forms of Cr, including nanoparticles. However, according to this organization using supplementation with Cr picolinate and yeast enriched with Cr in the amount of 0.8 mg Cr/kg of feed in poultry diet showed no effect on growth performance in chickens and turkeys [11]. The authors of various studies have supplemented the diet of poultry with chromium picolinate (Cr-Pic) in amounts from 200 to 4000 μg/kg and chromium nanoparticles (Cr-NP) in amounts from 200 to 1500 μg/kg, but as yet no toxic effect of these levels on the body has been confirmed [2, 6, 7].

Although numerous studies have established how Cr affects the metabolism, immunity, and growth performance of birds [2–4, 6], little is known about its accumulation in the intestines and other tissues, and especially about its effect on absorption of phosphorus in the enterocytes and its deposition in the tissues of chickens. Absorption of elements, including metal ions, in the small intestine depends on their chemical and physical form, but also on the presence of other substances in the diet, or on levels of certain hormones [12]. Chromium is probably absorbed in the enterocytes through channels transporting divalent metals such as iron, or via passive transport [12, 13]. The exact mechanism, however, is not yet known [11]. Chromium absorption has been shown to depend on its chemical form, i.e. Cr(VI) is better absorbed than Cr(III) [14]. Moreover, Cr in the form of complexes with organic ligands such as picolinate, propionate or amino acids (methionine, lysine, or histidine) is better absorbed in the gastrointestinal tract than Cr in the form of inorganic compounds such as CrCl_3_ [15, 16]. In recent years, scientific research has been focused on the use of Cr in the form of nanoparticles in poultry feeding due to their small size and potential more effective absorption [7, 17, 18]. Due to the fact that Cr in an organic form is absorbed better than in an inorganic form, it seems interesting to determine how Cr in the form of nanoparticles is absorbed and deposited in the body compared to the organic form of this element. The form of phosphorus (P), on the other hand, has a completely different effect on its absorption than in the case of Cr. Birds best absorb P in inorganic forms such as monocalcium phosphate, while organic forms such as phytates are not absorbed and require prior hydrolysis, e.g. by phytases [19–22]. Phosphorus is an important component of nucleic acids and phospholipids, and is also a cofactor for many enzymes [19]. Furthermore, it plays an important role in maintaining osmotic and acid-base balance, in protein synthesis, and in bone mineralization. It is a key mediator of energy metabolism through ATP [15]. Therefore, P deficiency can disrupt numerous biochemical processes in the cell and thus adversely affect growth performance.

Since Cr(III) is a cation, while P is an anion, these elements are presumed not to compete for enterocyte transport proteins. Once absorbed, Cr, by promoting the secretion of certain hormones, e.g. PTH, corticosterone, insulin, and dopamine [3, 5, 23], may indirectly influence the absorption of phosphorus, and thus its content in the cell and tissues. PTH regulates calcium and phosphate metabolism. This hormone mobilizes Ca from bone by activation of osteoclasts, resulting in the resorption of bone. When Ca level in blood is low, PTH also decreases phosphate reabsorption at the proximal convoluted tubule. When Ca level in blood is high, PTH increases phosphate reabsorption in kidney. According to McCarty [24] Cr probably diminishes PTH's ability to activate osteoclasts. Chromium increase insulin activity in bone, which can inhibit bone resorption by blocking the effect of PTH on osteoblasts, and more particularly, by impeding the PTH-mediated activation of protein kinase C (PKC).

There are reports that Cr accumulates in some tissues [25–27] and modifies the Ca metabolism in the body (including bones), so there is a supposition that it may also modify P metabolism (such studies have not been conducted so far). Disruption of P metabolism may result in deterioration of growth results, as a result of impaired deposition of this element in the bones as well as due to the dysfunction of some enzymes.

The aim of the study was to determine whether the form and level of Cr in the diet of chickens influences Cr accumulation in tissues as well as intestinal absorption of P and P deposition in tissues.

## Materials and methods

### Animals and diets

The experiment was carried out in a poultry house at the experimental facilities of the Department of Poultry Science, University of Warmia and Mazury, in Olsztyn, Poland. A total of 405 one-day-old male Ross 308 chickens were randomly divided into five groups, with nine replicates of nine birds each, and kept in cages. All birds had free access to feed and water. Each cage was equipped with nipple drinkers and a feeder that was manually filled on a daily basis. The heating and light program was in accordance with the Ross Broiler Management Manual [28]. All procedures involved handling the birds were performed by qualified veterinarians. No action involving pain or suffering was practiced, and all of the analyses were performed on samples collected post-mortem. The protocol for this study and the number of chickens used in this study were consistent with the regulations of the Local Committee for Experimentation on Animals (Olsztyn, Poland) and were performed in accordance with the principles of the European Union Directive 2010/63/EU for animal experiments and Polish Law on Animal Protection. The experimental procedure was approved by the Local Animal Experimentation Ethics Committee in Olsztyn (No. 30/2015).

The birds were fed a basal diet that was changed in two periods: starter (0–21 days) and grower/finisher (22–35 days) (Table 1). A control (C) group was fed the basal diet but without supplemental chromium. Experimental groups were fed basal diets supplemented with two levels of Cr (3 and 6 mg/kg) and two different Cr sources (Cr-picolinate–Cr-Pic and Cr-nanoparticles–Cr-NP). Four experimental diets were obtained: 3 mg/kg Cr-Pic, 6 mg/kg Cr-Pic, 3 mg/kg Cr-NP and 6 mg/kg Cr-NP. Chromium metal nanoparticles (Cr-NP, purity 99.9%, 60~80 nm, spherical, specific surface area 6–8 m^2^/g, bulk density 0.15 g/cm^3^, true density 8.9 g/cm^3^) was purchased from SkySpring Nanomaterials (USA). Chromium(III) picolinate (purity > 98%) was purchased from Sigma-Aldrich Sp. z o.o. (Poznan, Poland). The experimental additives were added to the feed mixtures in the form of suspensions in rapeseed oil (0.5%) on top of feed. Control group received 0.5% rapeseed oil without any additives on top of feed. All diets were isocaloric and isonitrogenous, and contained similar amounts of major amino acids (including lysine, methionine with cysteine, and threonine), minerals (including calcium and available phosphorus), and vitamins.

**Table 1:** Composition and nutrient density of diets.

	Starter	Grower/Finisher
	days 1–21	days 22–35
Components, g/kg		
Maize	200.0	200.0
Soybean meal	336.5	282.4
Wheat	383.4	421.4
Soybean oil	39.0	56.1
Salt	3.3	3.3
Limestone	11.9	11.6
Monocalcium phosphate	14.4	13.3
DL-Methionine	3.1	2.8
L-Lysine HCL	2.7	3.1
L-Threonine	0.7	1.0
Vitamins + trace minerals^1^	5.0	5.0
Calculated nutrient density, g/kg		
Crude protein	220.0	200.0
Lysine	13.0	12.0
Methionine	6.2	5.7
Met. + Cys.	10.0	9.2
Threonine	8.5	8.0
Calcium	9.5	9.0
Available phosphorus	4.8	4.5
ME, kcal/kg	2950	3100
Amount of Cr added to feed	Analysed content of Cr, mg/kg	
0	0.86	0.83
3 mg/kg Cr-Pic^2^	3.90	3.36
6 mg/kg Cr-Pic	6.71	6.20
3 mg/kg Cr-NP	3.85	3.87
6 mg/kg Cr-NP	6.49	6.08

^1^ Provided per kilogram of diet: days 1–21: vit. A, 15,000 IU; vit. D_3_, 5000 IU; vit. E, 112 IU; vit. K_3_, 4 mg; vit. B_1_, 3 mg; vit. B_2_, 8 mg; vitamin B_6_, 5 mg; vit. B_12_, 16 mg; folic acid, 2 mg; biotin, 0.2 mg; nicotinic acid, 60 m:; calcium pantothenate, 18 mg; choline, 1.8 g; Mn, 100 mg; Zn, 80 mg; Fe, 80 mg; Cu, 8 mg; I, 1 mg; Se, 0.15 mg; days 22–35: vit. A, 12,000 IU; vit. D_3_, 5000 IU; vit. E, 75 IU; vit. K_3_, 2 mg; vit. B_1_, 2 mg; vit. B_2_, 6 mg; vit. B_6_, 4 mg; vit. B_12_, 16 mg; folic acid, 1.75 mg; biotin, 0.05 mg; nicotinic acid, 60 mg; calcium pantothenate, 18 mg; choline, 1.6 g; Mn, 100 mg; Zn, 80 mg; Fe, 80 mg; Cu, 8 mg; I, 1 mg; Se, 0.15 mg

^2^Cr-Pic–chromium picolinate; Cr-NP–chromium nanoparticles

The nutritional value of all experimental diets corresponded to the nutrient requirements of broiler chickens [28].

### Sample collection

At 35 days of age, nine birds representing the average body weight of each group were selected, tagged, and fasted for 8 h. Blood samples were taken from the same nine birds from each group (one bird for each replication). Immediately after collection, blood samples were aliquoted into test tubes containing heparin as an anticoagulant. The samples were centrifuged for 15 min at 3000*g* and 4°C, and the obtained plasma was stored at −20°C until analysis.

Then, the same nine broilers per group (one bird representing the average body weight per pen) were killed at a slaughterhouse. The birds (without being transported) were electrically stunned (400 mA, 350 Hz), hung on a shackle line and exsanguinated by a unilateral neck cut severing the right carotid artery and jugular vein. After a 3-min bleeding period, the birds were scalded at 61°C for 60 s, defeathered in a rotary drum picker for 25 s, and manually eviscerated. Following evisceration, whole carcasses were stored at 4°C and hand-deboned on a cone 24 h post mortem. The carcasses were dissected, and samples of the ileum, liver, breast muscle, femur, skin, and feathers were collected. Ileum samples were used for analysis of P absorption.

### Ex vivo phosphorus absorption

Phosphorus absorption was tested using the *ex vivo* gastrointestinal sac technique described by Ognik et al. [29]. Immediately following removal of the small intestines, they were gently emptied of their contents and rinsed with physiological saline solution to remove feed residue. The anterior part of the intestine (about 20 cm of the ileum) was taken from nine birds in each experimental group and divided into a control segment (C) and experimental segments (E1 and E2). The jejunum area is easy to determine, so the ileum was collected at the border of the jejunum and ileum. Each of the 9 gut segments was divided into 3 smaller segments resulting an equal number of 9 gut segments for C, E1 and E2. Control segment was used for determination of Cr and P in ileum walls. Segments E1 and E2 were used to gut sacs preparation. The gut sacs were injected with 5 mL of a basal solution containing 4.8 g/L (E1) and 4.5 g/L of P (E2), depending on the period, in the form of H_2_PO_4_^2-^(absorption of P in phosphates is very often differentiated from 65 to 90% [30]): the amount of P in the basal solution was chosen based on available P content in the diet, i.e. 4.8 g/kg during the period of 1–21 d and 4.5 g/kg during the period of 22–35 d (Table 1). The sacs prepared in this manner were incubated for 2 h in a 100 mL serosal bath (0.9% NaCl) in a CO_2_ incubator at 37°C. Following incubation the sacs were rinsed with physiological saline and then cut open and dried at 60°C. For chemical analyzes, samples were taken in 3 replications from each segment (C, E1, E2).

### Determination of P and Cr content

The dried tissue samples for P determination were weighed to determine their dry weight. Then they were incinerated in a muffle furnace at 620°C for 5 h, and the ash was dissolved in 5 mL of 6 M HCl and diluted to 50 mL. Phosphorus contents in burnt tissue samples and in serosal bath were determined by a colorimetric assay measuring the reaction of phosphate ions with molybdate complexes in the presence of ascorbic acid solution [31]. Absorbance was measured in a spectrophotometer Thermo Scientific Genesis 20 at 660 nm. The plasma content of P was measured using an automatic biochemical analyser (Plasma Diagnostic Instruments Horiba, Kyoto, Japan).

For sample preparation for Cr determination, a 10 mL volume of concentrated HNO_3_ (Sigma Aldrich) was poured over weighed portions (usually 500±1 mg of each sample), which were then subjected to wet ashing. Mineralization was carried out in a Microwave Digestion System in Teflon vials (DAP 100), with optimal temperature and pressure applied to each individual sample, monitored throughout the acid digestion procedure (Bergh of Speedwave). Mineralization was performed according to the following scheme: 15 min with the temperature rising from room temperature up to 140°C, 5 min at a stable temperature of 140°C, 5 min with the temperature rising from 140°C up to 170°C, 15 min at 170°C and finally cooling down to room temperature (variable time). The pressure over the entire mineralisation process did not exceed 12 bar (1.2 MPa). A clear solution was obtained when the mineralisation process was completed. Next, the solution was cooled to room temperature and transferred to a 50 mL volumetric flask filled with demineralised water (ELGA Pure Lab Classic) to the 50 mL mark. Total concentrations of Cr in feed mixtures, plasma, ileum, liver, breast muscle, skin, thigh bones, and feathers were determined with an ICP-OES (Inductively Coupled Plasma Optical Emission Spectrometer) from Varian Inc., Palo Alto, CA, USA.

Cr intake was calculated based on DFI, BW and Cr content in feed and the equation:

$$Cr\;intake=\frac{DFI\;(1-14\;d)*Cr\;content\;in\;feed}{BW\;(14\;d)}+\frac{DFI\;(15-35\;d)*Cr\;content\;in\;feed}{BW\;(35d)}$$

### Statistical analysis

The Statistica software package version 13.1 (Statsoft Inc., 2016) was used to determine whether variables differed between treatment groups. Values below the limit of quantification (LOQ) in the statistical analysis were set as “0”. The comparison of control group vs all other groups was performed by planned contrast analysis. Two-way ANOVA was performed to assess the effects of the chromium supplementation levels, the source of chromium, and the interaction between the level and source (level x source). When the ANOVA indicated significant treatment effects, means were separated using Tukey’s multiple range test. The results are presented in the tables as means with pooled standard errors. The residuals were checked for normality using Shapiro-Wilk test prior to the statistical analysis. Differences were considered significant at *P* ≤ 0.05.

## Results

Compared to the control group, both 3 and 6 mg/kg Cr added to the diet of chickens, irrespective of its form, led to higher intake of Cr (*P* < 0.001). An increase in Cr content in ileum was noted in group receiving 6 mg/kg Cr-Pic and 3 mg/kg Cr-NP relative to control group (*P* = 0.025). Compared to the control group, in liver of chickens receiving 6 mg/kg Cr-Pic and Cr-NP irrespective of its level, higher Cr content was noted (*P* = 0.013). An increase in Cr content in breast muscle was noted in group receiving 6 mg/kg Cr-NP (*P* = 0.048), while an increase in Cr content in skin was determined in group receiving 6 mg/kg Cr-Pic (*P* = 0.038). Compared to the control group, both 3 and 6 mg/kg Cr added to the diet of chickens, irrespective of its form resulted in increased content of this element in feathers (*P* < 0.001). An increase in Cr content in femur was noted in group receiving Cr-Pic irrespective of its level relative to control group (*P* = 0.042) (Table 2).

**Table 2:** Cr content in selected tissues and feathers.

Treatment^1^	Cr intake	Ileum	Liver	Breast muscle	Femur	Skin	Feathers
	mg/kg BW	μg/g	μg/g	μg/g	μg/g	μg/g	μg/g
Control	2.20	0.206	0.454	0.168	0.309	0.224	<LOQ
3 mg/kg Cr-Pic	9.62*	0.247^b^	0.498	0.176^bc^	0.524^b^*	0.219	0.635^b^*
6 mg/kg Cr-Pic	16.97*	0.389^a^*	0.511*	0.184^b^	0.959^a^*	0.286*	1.350^a^*
3 mg/kg Cr-NP	10.44*	0.356^ab^*	0.579*	0.170^c^	0.316^c^	0.229	0.545^c^*
6 mg/kg Cr-NP	16.46*	0.193^c^	0.590*	0.230^a^*	0.315^c^	0.255	0.589^c^*
SEM	0.362	0.019	0.023	0.014	0.046	0.038	0.052
Level (L)							
3 mg/kg	10.03	0.302	0.538	0.173	0.420	0.224	0.590
6 mg/kg	16.72	0.291	0.551	0.207	0.637	0.271	0.969
Source (S)							
Cr-Pic	13.29	0.281	0.504	0.180	0.714	0.253	0.993
Cr-NP	13.45	0.252	0.585	0.200	0.316	0.242	0.567
*P-value*							
Control vs. all others	<0.001	0.025	0.013	0.048	0.042	0.038	<0.001
Level (L)	<0.001	0.181	0.026	<0.001	<0.001	<0.001	<0.001
Source (S)	0.144	0.009	<0.001	<0.001	<0.001	0.024	<0.001
L x S interaction	0.245	<0.001	0.820	<0.001	<0.001	0.302	<0.001

^a,b,…^ Means within the same column differ significantly (*P* ≤ 0.05) according to Tukey mean comparison (only in the case of significant L×S interaction). SEM = standard error of the mean (SD for all chickens divided by the square root of the number of chickens, n = 45).

^1^Cr-Pic–chromium picolinate; Cr-NP–chromium nanoparticles

LOQ- limit of quantification

Irrespective of source, an increase in Cr content in liver (*P* = 0.026) and skin (*P* < 0.001) were noted in chickens receiving 6 mg Cr/kg compared to chickens receiving 3 mg Cr/kg. The addition of Cr in the form of Cr-Pic increased the accumulation of this element more than the addition of Cr-NP in the skin (*P* = 0.024). On the other hand, chickens from the Cr-NP treatment had higher Cr content in the liver (*P* < 0.001) than chickens from the Cr-Pic treatment (Table 2). Two-way ANOVA showed level × source interactions for Cr content in the ileum (*P* < 0.001), breast muscle (*P* < 0.001), femur (*P* < 0.001) and feathers (*P* < 0.001). The interaction was due to the fact that the addition of 6 mg/kg of Cr-Pic to diet compared to addition of 3 mg/kg of Cr-Pic increased Cr content in ileum of the chickens, while the corresponding level of Cr-NP decreased it. On the other hand, the addition of 6 mg/kg of Cr-NP to diet compared to addition of 3 mg/kg Cr-NP increased Cr content in the breast muscle, which was not observed for Cr-Pic. The addition of 6 mg/kg of Cr-Pic compared to addition of 3 mg/kg of Cr-Pic resulted in an increase in Cr content in the femur and feathers, while this effect was not observed for Cr-NP (Table 2).

In our study, the ex vivo test showed no effect of deposited Cr in the ileum wall on P absorption. Similarly, the use of Cr in the diet in the form of both Cr-Pic and Cr-NP had no effect on estimated intestinal absorption of P (Table 3).

**Table 3:** Concentration of P in the serosal bath at 4.5 and 4.8 g P/L in the gut sac.

Treatment^1^	Concentration of P mg/L in serosal bath at 4.5 g P/L in gut sac	% P in serosal bath at 4.5 g P/L in gut sac	Concentration of P mg/L in serosal bath at 4.8 g P/L in gut sac	% P in serosal bath at 4.8 g P/L in gut sac
Control	4.138	91.95	4.159	86.64
3 mg/kg Cr-Pic	4.121	91.57	4.146	86.37
6 mg/kg Cr-Pic	4.140	92.00	4.146	86.38
3 mg/kg Cr-NP	4.140	91.99	4.175	86.97
6 mg/kg Cr-NP	4.152	92.26	4.153	86.51
SEM	0.123	0.118	0.144	0.203
Level (L)				
3 mg/kg	4.130	91.78	4.160	86.67
6 mg/kg	4.146	92.13	4.149	86.44
Source (S)				
Cr-Pic	4.130	91.78	4.146	86.37
Cr-NP	4.146	92.13	4.164	86.74
*P-value*				
Control vs. all others	0.214	0.354	0.158	0.462
Level (L)	0.326	0. 214	0.236	0.415
Source (S)	0.102	0.177	0.132	0.105
L x S interaction	0.141	0.423	0.126	0.107

^a,b,…^ Means within the same column differ significantly (*P* ≤ 0.05) according to Tukey mean comparison (only in the case of significant L×S interaction). SEM = standard error of the mean (SD for all chickens divided by the square root of the number of chickens, n = 45).

^1^Cr-Pic–chromium picolinate; Cr-NP–chromium nanoparticles

Compared to the control group, both 3 and 6 mg/kg Cr added to the diet of chickens, irrespective of its form, led to higher P content in ileum and skin (*P* = 0.041 and *P* = 0.026, respectively). An increase in P content in blood plasma and feathers was noted in group receiving 6 mg/kg Cr-Pic and 3 mg/kg and 6 mg/kg Cr-NP relative to control group (*P* = 0.038; *P* = 0.037, respectively). Compared to the control group, in groups 6 mg/kg Cr-Pic and 3 mg/kg and 6 mg/kg Cr-NP, in femur lower P content was noted (*P* = 0.012). An increase in P content in liver was noted in group receiving Cr-Pic irrespective of its level (*P* = 0.046), while decrease in P content in blood plasma was determined in group receiving 3 mg/kg Cr-Pic (*P* = 0.038) compared to control group (Table 4).

**Table 4:** Content of phosphorus in selected tissues and feathers.

Treatment	Blood plasma	Ileum	Breast muscle	Liver	Femur	Skin	Feathers
	mmol/L	g/kg	g/kg	g/kg	g/kg	g/kg	g/kg
Control	1.826	1.882	1.092	2.321	80.63	0.324	<LOQ
3 mg/kg Cr-Pic	1.546^c^*	2.020^b^*	0.962	2.766^a^*	81.75	0.442*	<LOQ^d^
6 mg/kg Cr-Pic	2.151^b^*	2.580^a^*	1.084	2.081^c^*	51.51*	0.697*	0.062^c^*
3 mg/kg Cr-NP	2.296^a^*	2.110^b^*	1.134	2.260^b^	60.63*	0.599*	0.289^a^*
6 mg/kg Cr-NP	2.254^a^*	2.081^b^*	1.186	2.279^b^	50.33*	0.763*	0.161^b^*
SEM	0.078	0.015	0.023	0.075	0.590	0.055	0.031
Level (L)							
3 mg/kg	1.921	2.065	1.048	2.513	71.17	0.520	0.289
6 mg/kg	2.202	2.330	1.135	2.180	50.90	0.730	0.111
Source (S)							
Cr-Pic	1.848	2.300	1.023	2.423	66.63	0.509	0.062
Cr-NP	2.275	2.096	1.160	2.269	55.47	0.681	0.225
*P-value*							
Control vs. all others	0.038	0.041	0.132	0.046	0.012	0.026	<0.001
Level (L)	0.009	0.009	0.037	<0.001	<0.001	<0.001	<0.001
Source (S)	0.035	0.035	0.009	0.018	<0.001	0.005	<0.001
L x S interaction	0.005	0.005	0.472	<0.001	0.529	0.266	<0.001

^a,b,…^ Means within the same column differ significantly (*P* ≤ 0.05) according to Tukey mean comparison (only in the case of significant L×S interaction). SEM = standard error of the mean (SD for all chickens divided by the square root of the number of chickens, n = 45).

^1^Cr-Pic–chromium picolinate; Cr-NP–chromium nanoparticles

Irrespective of source, an increase in P content in breast muscle (*P* = 0.037) and skin (*P* < 0.001) and decrease in P content in femur (*P* < 0.001) were noted in chickens receiving 6 mg Cr/kg compared to chickens receiving 3 mg Cr/kg. The addition of Cr to the diet in the form of Cr-NP increased phosphorus levels more than in the form of Cr-Pic in the breast muscle (*P* = 0.009), and skin (*P* = 0.005), while causing a greater decrease in the femur (*P* < 0.001).

The results of two-way ANOVA showed level × source interactions for P content in the blood plasma (*P* = 0.005), ileum (*P* = 0.005), liver (*P* < 0.001) and feathers (*P* < 0.001; Table 4). The analysis showed that the addition 6 mg/kg of Cr-Pic to diet compared to the addition of 3 mg/kg of Cr-Pic increased P content in the blood plasma and ileum, which was not observed for Cr-NP. The addition of 6 mg/kg of Cr-Pic to diet compared to 3 mg/kg Cr-Pic decreased P content in the liver, which was not observed for Cr-NP. On the other hand, the addition of 6 mg/kg of Cr-Pic compared to 3 mg/kg of Cr-Pic increased the content of P in the feathers, while the addition of 6 mg/kg of Cr-NP to diet compared to 3 mg/kg of Cr-NP decreased it (Table 4).

## Discussion

Cr is absorbed in the small intestine together with other metal ions [11], but the exact mechanism of this process is not yet fully understood. Cr(III) absorbed in the intestine binds to plasma proteins, which transport it to the liver and other organs. The transport protein is usually transferrin [25]. Our study shows that the addition of Cr to the diet of chickens depending on the source increased its accumulation in ileum, liver, breast muscle, femur, and especially in the feathers. However, increasing the dose of Cr from 3 to 6 mg/kg increased the accumulation of this element in the selected tissues and feathers. Many researchers have found that Cr accumulates mostly in the liver, kidneys, and spleen, and less in the heart, muscles, bones, and brain [25–27]. Sirarat et al. [7] reported that Cr administered to broilers in the form of Cr-Pic nanoparticles accumulates mainly in the liver of the birds. Some tissues, such as bone, testicular, and epididymal tissues, have been shown to accumulate Cr over a long period of time, whereas accumulation of this element in the heart, pancreas, and brain is relatively short-lived [32]. Cr is probably incorporated into the bone structure in the mineralization process. Due to their similar ionic radius, Cr can probably replace Ca in bones, thereby affecting their structure. According to literature data [20–22], Ca content in chicken bones is about 20%. Therefore, based on our research, it can be assumed that a very small amount of Cr which was deposited in the femur (from about 0.0003% in the control group to about 0.0006% for the 6 mg/kg supplement) can potentially be integrated into the bone structure in place of Ca. According to Bronner [33], the total Cr content in bones is about 2.5 times as high as in all other tissues. Accumulation of Cr in the skeleton does not seem desirable, as it may change the structure of hydroxyapatite and thus increase bone fragility. Our study shows that in addition to the level of Cr used, its form also affects its accumulation in tissues. Chromium applied in the form of Cr-Pic accumulates more in the femur, skin and feathers than Cr in the form of Cr-NP, while in the form of Cr-NP it accumulates more in the liver and breast muscle than in the form of Cr-Pic. In contrast, Sathyabama et al. [34], in research conducted on laying hens, found that Cr accumulation in tissues was not affected by the form used (Cr-Pic or Cr-NP), but only by the level applied. Cr(III) administered orally as either inorganic salt or an organic complex was not acutely toxic to rats, with LD_50_ > 2000 mg/kg BW [9]. Rhodes et al. [35] reported a no-observed-adverse-effect level (NOAEL) ≥ 2015 mg/kg BW/day for chromium picolinate monohydrate. Therefore, even in the case of the highest Cr intake by chickens, i.e. about 17 mg/kg BW, neither LD_50_ nor NOAEL was exceeded. Excessive accumulation of Cr in the liver may lead to disturbances in liver metabolism, intensify oxidative reactions and lead to morphological changes in this organ [2, 8].

The available literature provides no data on the mechanism of Cr absorption in enterocytes, whereas the mechanism of P absorption has been described quite thoroughly. Absorption of P in the small intestine can be paracellular, taking place through passive diffusion or active transport involving Na-dependent transport proteins [36]. The most common is transcellular absorption, which involves three phosphate co-transporters: type IIb Na-dependent phosphate co-transporter (NaPi-IIb), inorganic phosphate transporter 1 (PiT-1), and inorganic phosphate transporter 2 (PiT-2). NaPi-IIb in broilers is primarily expressed in the duodenum and is considered the most important P transporter in the small intestine [36]. According to Hu [37] PiT-1 and PiT-2 are expressed in the small intestine of broilers, and also regulate intestinal P absorption.

Our ex vivo tests showed that the use of Cr in the diet of chickens in various forms and levels would not affect intestinal absorption of P. The results can probably be explained by the fact that Cr and P have different ion charges and can therefore be absorbed into enterocytes through different channels and may have different affinities to transport proteins.

Nevertheless, Cr absorbed into the body can indirectly affect the P level in plasma and its deposition in other tissues. However, it should be noted that the effect of dietary Cr on P absorption can be dependent on age (young chickens have an immature intestinal epithelium compared to 35 days old birds). P level in blood plasma is dependent on time of slaughtering, and changes over the day with distance from last feeding. Literature data indicate that P content in chicken tissues is about 20 mg/L in plasma [37], from 0.116 g/kg [37] to 1.32 g/kg [7] in the liver, from 0.108 mg/kg [37] to 1.7 g/kg [38] in muscles, and from 0.292 g/kg [37] to 83 g/kg [16–18, 39] in the bones. Sirirat et al. [7], in a study in chickens receiving supplements of 500 or 3000 ppb Cr in the form of nanoparticles, noted a higher content of P in the liver (by 67% compared to the control). In contrast, Sathyabama et al. [34] reported no effect of dietary Cr supplementation on plasma or liver levels of P in laying hens. Our study showed that as the Cr level in the diet of chickens increased, irrespective of the form used, P levels increased in the ileum, blood plasma, breast muscle, skin, and feathers, but decreased in the liver and femur. According to Sahin et al. [4], Cr levels and forms used in the diet of chickens may have reduced the secretion of corticosterone, a hormone associated with stress. Sahin et al. [4] noted reduced plasma corticosterone levels in chickens exposed to heat stress and at the same time receiving Cr in the diet. Available literature data show that corticosterone reduces absorption of phosphates into the enterocytes and then into the bloodstream [23], but this effect was not noted in our study. Intestinal absorption and accumulation of phosphates in tissues may also be regulated by other hormones, including PTH, insulin, and dopamine, whose secretion is influenced by Cr [23, 40]. Our previous research [5] has shown that the addition of Cr to the diet of chickens in the form of both Cr-Pic and Cr-NP increases insulin secretion, while the addition of Cr in the form of Cr-NP increases plasma dopamine levels.

According to Sankaramanivel et al. [41], phosphorus is unlikely to be released from bones; if the level is reduced in cells, it is more likely that absorption from the intestine will increase or that its elimination in the urine will decrease. Our research, however, indicates that resorption of P from bones was very high (at about 30%) after application of Cr-NP (3 and 6 mg/kg) and Cr-Pic at 6 mg/kg compared to the control, which is an adverse effect of the use Cr in the diet of chickens. This is an innovative result of our research, as it indicates the potential risk of using Cr in the diet of chickens. Recent research has shown that bone mineralization is also affected by oxidative stress [42]. According to Soudani et al. [42], Cr(VI) is a well-known oxidizing agent in several tissues. Intracellular Cr(VI) is converted to more stable Cr(III) with the production of reactive oxygen species (ROS). According to Yang et al. [43] and Sontakke and Tare [44], ROS are involved in the resorption of Ca and P from bone. Our previous research [5] showed that Cr may have a pro-oxidative effect, and therefore the reduction in P levels noted in the bones may also be associated with oxidative stress generated by Cr. Stępniowska et al. [5] noticed that the addition of 6 mg/kg Cr to the diet of chickens, increased lipid peroxidation in both the liver and breast muscle, as evidenced by increased LOOH levels. Also the changes in the activity of antioxidant enzymes noted in this study were the body’s response to the intensification of oxidative processes due to the addition of Cr to the diet. Additionally the addition of Cr to the diet of chickens in the form of both Cr-Pic and Cr-NP adversely affects growth performance. Chickens receiving Cr supplements had lower final body weights and higher daily feed intake [2]. Therefore, this research should be continued to enable a detailed assessment of the absorption, digestibility, and deposition of P, Ca and Cr in chicken tissues.

## Conclusions

Cr was found to accumulate in the tissues of the ileum, liver, breast muscle, and in feathers of chickens. The amount of Cr deposited in the tissues increases with the addition of Cr to the diet, irrespective of the form used. Chromium in the form of Cr-Pic is accumulated more in bones, skin and feathers, while in the form of Cr-NP it is accumulated more in the liver and breast muscle. Chromium deposited in the ileum of chickens does not affect the ex vivo estimated intestinal absorption of P.

The addition of 6 mg Cr/kg to the chicken’s diet more than 3 mg Cr/kg increased the content of P in the ileum, blood plasma, breast muscle, and skin, while adversely decreasing it in the liver, femur, and feathers. Cr reduced the amount of P in the femur when used in the form of Cr-NP at just 3 mg/kg, and in the form of Cr-Pic at 6 mg/kg.
